# Comparing a ses-sensitive and an all-ses implementation strategy to improve participation rates of patients with a lower socioeconomic background in a web-based intervention for depressive complaints: a cluster randomised trial in primary care

**DOI:** 10.1186/s12875-022-01793-w

**Published:** 2022-08-11

**Authors:** Stephanie S. Leone, Odile Smeets, Suzanne Lokman, Brigitte Boon, Agnes van der Poel, Tessa Van Doesum, Laura Shields-Zeeman, Jeannet Kramer, Filip Smit

**Affiliations:** 1 Department of Mental Health & Prevention, Trimbos Institute: Netherlands Institute of Mental Health and Addiction, Utrecht, Netherlands; 2 Academy Het Dorp, Arnhem, The Netherlands; 3 Tranzo, Tilburg School of Social and Behavioral Sciences, Tilburg University, Tilburg, The Netherlands; 4 Siza, Arnhem, The Netherlands; 5 Department of Epidemiology and Biostatistics and Department of Clinical, Neuro and Developmental Psychology, Public Health Research Institute, University Medical Center Amsterdam, Amsterdam, Netherlands

**Keywords:** E-health, Implementation, Depression, Primary Care, Lower socio-economic status

## Abstract

**Background:**

Depression is a major public health concern, which is most pronounced in population segments with a lower social-economic status (SES). E-health interventions for depressive complaints are proven to be effective, but their reach needs to be improved, especially among people with a lower socioeconomic status (SES). Implementing e-health interventions in the primary care setting with SES-sensitive guidance from General Practice nurses (GP nurses) may be a useful strategy to increase the reach of e-health in lower SES groups. We implemented an evidence-based online intervention that targets depressive complaints in primary care.

**Methods:**

A pragmatic cluster-randomised trial was conducted in two parallel groups where a SES sensitive (SES-sens) implementation strategy with additional face-to-face guidance by GP nurses was compared to an all-SES implementation strategy. The primary outcome was the percentage of lower SES participants in either condition. Participation was defined as completing at least 1 face-to-face session and 2 online exercises. Participation rates were evaluated using logistic mixed modelling.

**Results:**

In both conditions, the participation rates of lower SES participants were quite high, but were notably lower in the SES-sens implementation condition (44%) than in the all-SES implementation condition (58%). This unexpected outcome remained statistically significant even after adjusting for potential confounders between the conditions (Odds Ratio 0.43, 95%-CI 0.22 to 0.81). Less guidance was provided by the GP nurses in the SES-sens group, contrary to the implementation instructions.

**Conclusions:**

From a public health point of view, it is good news that a substantial number of primary care patients with a lower SES level used the implemented e-health intervention. It is also positive that an all-SES implementation strategy performed well, and even outperformed a SES-sensitive strategy. However, this was an unexpected finding, warranting further research into tailoring implementation strategies of e-health interventions towards specific target groups in the primary care setting.

**Trial registration:**

Netherlands Trial Register, identifier: NL6595, registered on 12 November 2017.

**Supplementary Information:**

The online version contains supplementary material available at 10.1186/s12875-022-01793-w.

## Background

Depressive disorder is a major public health concern [1–3] and there is increasing evidence showing that interventions aimed at preventing and reducing depressive symptoms are effective including internet-based (self-help) interventions [4, 5]. Nevertheless, the reach of preventive interventions is modest, and internet-based preventive interventions have been proposed as a potential solution due to their advantages over regular (face-to-face) care such as increased accessibility, flexibility, scalability and expected cost-effectiveness [6, 7]. However, users of internet-based interventions are more likely to have a high level of education [8]. This is a concern, as prior research shows that people with a lower SES are particularly at risk for depression [9]. Implementing (preventive) online interventions in routine care with guidance from a health care professional (i.e. in ‘blended’ format) may offer a strategy for increasing the reach among lower SES population segments and may improve adherence and effectiveness [10–13]. In the Netherlands, treatment of mild to moderate mental health complaints is primarily a task of the general practitioner (GP). The GP mental health nurse (GP nurse) has an important role in delivering mental healthcare in the GP setting and increasingly offers e-health interventions [14, 15]. However, GP nurses have identified barriers of implementing e-health such as the lack of suitable e-health interventions (particularly for lower educated patients), the inability of available interventions to be tailored to the patient’s personal complaints, early dropout of patients, and not having enough opportunity to familiarise themselves with online interventions [15].

The online complaint-directed mini-interventions (CDMIs) are brief unguided web-based interventions that target depressive complaints by focussing on highly prevalent complaints associated with depression, which impede daily functioning, and are associated with increased healthcare costs: stress, sleep problems and worry [16]. The unguided CDMIs were found to be effective and cost-effective in reducing depressive complaints [17, 18] and are widely available in The Netherlands. The CDMIs allow patients to select the complaint(s) they want to focus on and pick and mix the CDMI modules. Exercises offered by the online CDMIs were designed to appeal across social and economic groups. However, 70% of participants in our prior trial were highly educated, corroborating demographic data in previous research [5, 8].

This demonstrates the need to identify effective implementation strategies to increase the uptake of effective intervention among people who may be at greatest risk of depression. This study reports on the findings of a pragmatic cluster-randomised controlled trial to evaluate the implementation of the CDMIs in primary care and determine whether a SES-sensitive implementation strategy improves the participation rate of lower-SES patients.

## Methods

### Design

**Fig. 1:**
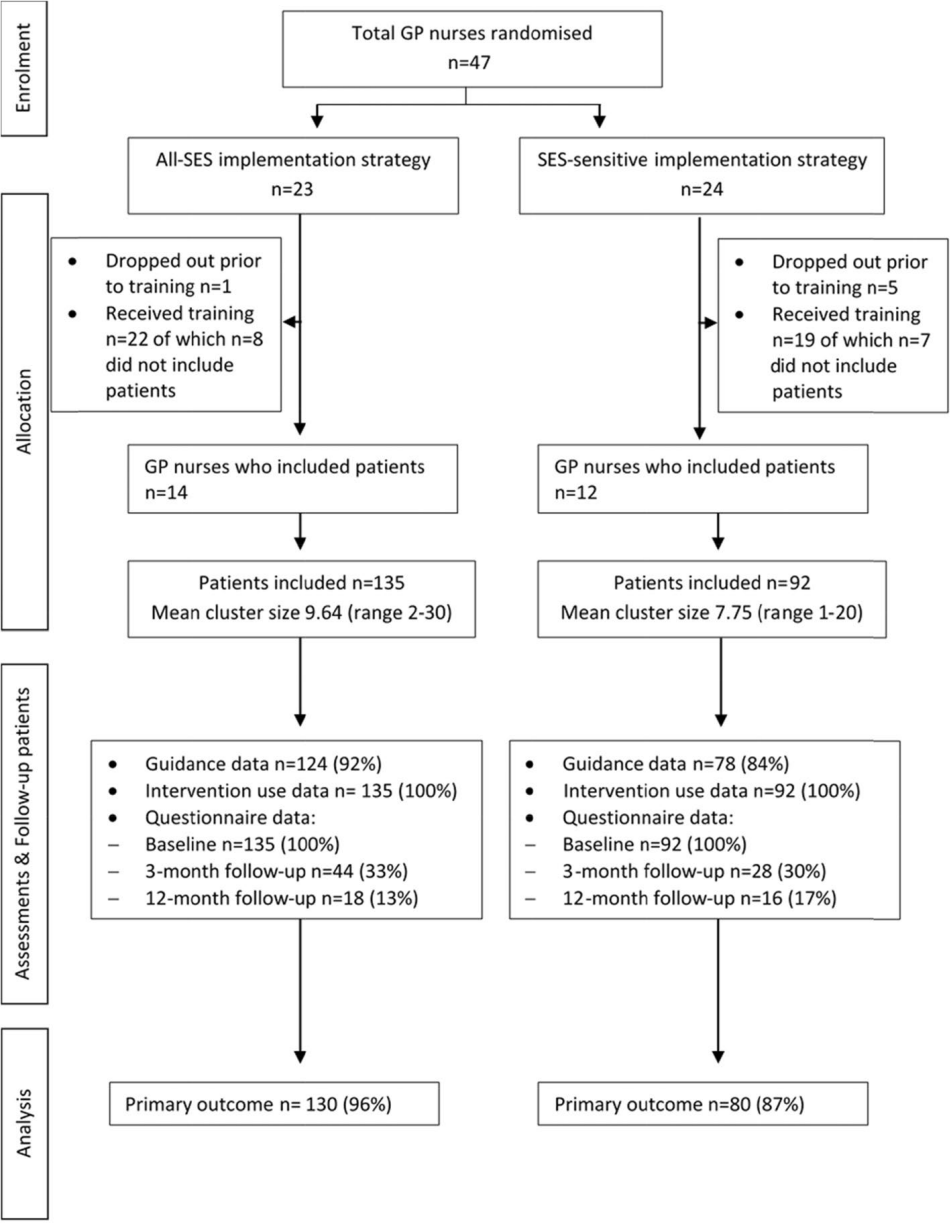
Flowchart of study

We conducted a pragmatic cluster-randomised controlled trial with two parallel groups comparing a SES sensitive implementation strategy (SES-sens condition) for implementing the online CDMIs to an implementation strategy that is not tailored to a specific SES group (all-SES condition). We hypothesised that the percentage of lower-SES participants in the online CDMIs would be higher in the SES-sens condition as compared to the all-SES condition. As the GP nurse plays a key role in the implementation strategies, randomisation took place at the level of the GP nurse in order to avoid contamination between the two implementation strategy conditions. The study protocol has been described elsewhere [19]. In brief, participating patients had access to the online CDMIs in both conditions and were permitted to receive any other type of care during this pragmatic trial. Patients were assessed at baseline (T0), and at 3 months (T1) and 12 months post baseline (T2), while participating GP nurses were assessed at baseline, and 6 months after baseline. Figure 1 shows the study flowchart in accordance with the Consort Statement for cluster randomised trials [20]. Moreover, this study is described in accordance with the Consolidated Standards of Reporting Trials (CONSORT) Statement and checklist (Additional file 1) [20]. Ethical approval for the study was granted by the VU Amsterdam Medical Center Ethics Committee (reference number 2017.437) and is registered in the Netherlands Trial Register (NL6595, registration date 12 November 2017, https://www.trialregister.nl/trial/6595).

### Participants

GP nurses from two primary care organisations serving various GP practices in two different regions (Rotterdam and Almere) in the Netherlands participated in the study. Participating GP nurses recruited participants among their current case load according to the implementation strategy to which they were randomly assigned. Patients were recruited between January 2018 and June 2019. Patients were eligible for study inclusion if they:

- Were 18 years of age or older
- Experienced worry, stress or sleep problems (determined by the GP nurse)
- Had Internet access
- Had sufficient proficiency of the Dutch language
- Had no acute or urgent comorbidity
- Provided informed consent.

Suicidal ideation was not considered a criterium for exclusion in view of the recently published report about the risk of including suicidal patients in RCTs [21]. To be able to provide suitable care, suicidal thoughts were assessed in the CDMI monitoring system (see below) at baseline, and 3 and 12 months using item 15 of the Web Screening Questionnaire (WSQ) [22].

### Inclusion procedure

The GP nurse informed eligible patients about the online CDMIs and the study during a consultation and by sending them a digital invitation by email with information about the study and a link to the online informed consent form. Patients provided informed consent by clicking on the ‘I consent’ button. After giving consent, patients created their own secure online CDMI account. After logging in they were asked to complete the online baseline questionnaire. See Fig. 1 for the flowchart.

### Implementation strategies

The implementation strategies were based on the implementation model of Grol & Wensing [23]. For a detailed description of the development process see [19]. At the start of our study there were no implementation-as-usual strategies for e-mental health in primary care. Therefore, we designed a general implementation strategy (all-SES), based on theory, evidence and practice, which did not place any emphasis on reaching and guiding lower SES patients. In addition, we designed a SES-sensitive implementation strategy which shares the same set of (sub-)strategies as the all-SES strategy, but some are specifically tailored to be SES-sensitive. To facilitate implementation, the online CDMIs were linked to a monitoring system which provided the GP nurse with insight into the intervention use of their patients (e.g. answers to questionnaires, diary entries, number of logins, number of completed exercises), and allowed them to send messages to their patients for coaching and encouragement.

#### All-SES implementation strategy

GP nurses received a 2-h training in which they: 1. learned about the online CDMIs, the related monitoring system and guidelines for the recruitment and guidance of patients through the CDMIs, 2. practiced using the online CDMIs and its monitoring system, and 3. discussed possible barriers and facilitators for implementation. The GP nurses were provided with an implementation manual containing information about blended e-health interventions, the content of the online CDMIs, the monitoring system, the recruitment and guidance process, the study flowchart and patient information. General guidelines were provided on topics that could be discussed with patients and on how to tackle potential barriers in the patient’s motivation to initiate and continue using the CDMIs.

GP nurses were free to choose the amount and type of guidance they provided to patients in working through the CDMI’s, but 3 consultations were recommended: at the start, mid-point and end of the patient using the CDMI. The only requirement was to have at least a single face-to-face consultation.

The research team provided ongoing support to the GP nurses throughout the implementation period: GP nurses received follow-up training sessions and could participate in 3 implementation team meetings, to exchange experiences and lessons learned, and discuss implementation barriers. The research team provided a helpdesk service (by email or telephone) for any technical or implementation-related queries. To keep the CDMI’s on the agenda of GP nurses, the research team communicated regularly through newsletters, telephone calls and emails with information and tips for the CDMIs. For patients, the all-SES strategy consisted of: (1) receiving a brochure with information about the online CDMIs (what are the CDMIs for, how to use them, option to have guidance by GP nurse), including information about the study; (2) receiving reminders to use the online CDMIs after a lack of activity; and (3) receiving support from the GP nurse in accordance with guidance outlined in the implementation manual.

#### SES-sensitive implementation strategy

The topics described in the all-SES strategy were identical in the SES-sens strategy, but were further tailored to better meet the needs of the lower-SES target group. To that end, the GP nurses randomised to this strategy received a 3-h training in which extra elements of the SES-sens strategy were addressed. This included the advice to use a more proactive approach [24] to involve and guide patients in using the CDMIs as this was expected to be more beneficial. Also, GP nurses learned about the consequences of low health literacy skills, that is more problems with: finding their way in healthcare; searching the web; understanding texts; communicating with healthcare providers and self-management. GP nurses were taught concrete strategies for communication (for instance, ‘use short sentences in the present tense’) and for guidance (for instance ‘do not give a writing assignment’ and ‘ask open-ended questions’). This was deemed important especially since limited health-literacy skills are more prevalent among vulnerable groups, including those with low education and low income [25–27]. The GP nurses also had access to a helpdesk service, three implementation team meetings and received communication from the research teams, with special attention to the lower-SES target group in all these actions. The additional elements of the SES-sens strategy aimed at the patients were: receiving extra guidance from the GP nurse where needed, e.g. with the registration process and when working through the exercises.

## Outcomes

### Primary outcome

The primary outcome was the difference between the all-SES and the SES-sens groups in the participation rate (i.e. the proportion) of patients with a lower SES. Participation was defined as participating in at least one face-to-face session with a GP nurse (registered retrospectively by the GP nurse) and engaging in at least two CDMI exercises (determined from user data from the online CDMIs). We hypothesised that the proportion of patients with a lower SES would be twice as high in the SES-sens group compared to the all-SES group.

#### Lower SES status

Though SES can be operationalised in various ways, education level, income and employment status/ occupation are often used as indicators of SES, and all three were used in our study [28–32] (see [19] for a detailed description of the rationale). A lower SES status was defined as:

1. Having an intermediate vocational education (in Dutch: MBO) or lower as the highest completed educational level (main indicator of SES), *and/or*
2. Being unemployed and living in a neighbourhood with a negative SES level score, *and/or*
3. Having a total gross family income of below the social minimum income in the Netherlands (see below for thresholds), and living in a neighbourhood with a low social status level score.

Self-reported education was assessed with a single item (*What is your highest completed educational level?*) and categorised as: none/primary school, lower vocational education, intermediate secondary education, higher secondary education, intermediate vocational education, higher vocational education, academic education. In the Netherlands, four levels of intermediate vocational education are discerned, and generally the first level is used as a cut-off for low SES. A higher cut-off (i.e. level 4) is used in this study, as mostly highly educated patients (i.e. higher vocational education and academic education) are currently reached with e-health interventions. Thus, we use the term ‘lower SES’.

Social minimum income was assessed using self-reported gross family income (single item) and self-reported living arrangements. In the Netherlands, the social minimum income is dependent upon living arrangements and defined as a minimum income of an average of €1100 per month for persons who live alone or as a single parent, and average €1550 for persons with any other type of living arrangement.

Self-reported work status was assessed with a single item (employed/self-employed, unemployed, occupationally disabled, student, volunteer work, retired, homemaker).

Social status level scores of the neighbourhood were assessed by using the self-reported four-digit zip code and matching it to the zip code’s corresponding SES score as provided by The Netherlands Institute for Social Research (for the year 2016). A low SES score indicates a lower-than-average social status level.

The components of the primary outcome were either assessed at T0 (i.e. lower SES status) or during the intervention period (i.e. number of completed exercises and face-to-face sessions).

### Secondary outcomes

#### Psychological complaints and wellbeing

Psychological complaints were assessed at T0, T1 and T2. *Depressive complaints* were measured using the 8-item Patient Health Questionnaire (PHQ-8) [33] Scores can range from 0 to 24 with higher scores indicating higher levels of depressive complaints. *Sleep problems* were measured with the 4-item Jenkins Sleep Evaluation Questionnaire (JSEQ) [34], with a total score range from 0 to 20. Higher scores indicate more sleep problems. *Stress* was measured with the 10-item Perceived Stress Scale (PSS-10) [35] with scores ranging from 0 to 40 and higher scores indicating higher stress levels. *Worry* was assessed using the 11-item Penn State Worry Questionnaire (PSWQ) [36], with a score range from 11 to 55 and higher scores indicating more worry. *Anxiety* was measured with the 7-item Generalised Anxiety Disorder Scale (GAD-7) [37], resulting in a total score range of 0–28, with higher scores indicating a higher level of anxiety severity. *Well-being* was assessed with the 5-item World Health Organisation Well-Being Index (WHO-5) [38]. The total score can range from 0 to 100. Higher scores correspond to higher levels of well-being.

#### Health literacy

Health literacy was assessed at baseline using the 14-item Dutch Functional Communicative and Critical Health Literacy Scale (FCCHL) [39, 40]. The mean total score can range from 1 to 4, with higher scores indicating higher levels of health literacy.

#### Process indicators of implementation

Patients were asked about the perceived utility of and satisfaction with the intervention (e.g. ease of use, effectiveness in reducing complaints, relevance of exercises, and satisfaction overall) at 3- and 12-month follow-up. At 3-month follow-up, patients were asked about the utility of, and satisfaction with the guidance offered by the GP nurses. In-depth interviews (*n* = 9) were conducted to elicit perceived barriers and facilitators of using the (blended) CDMIs alongside open-ended questions in the questionnaire.

At baseline GP nurses were asked to rate statements (1 = totally agree, 5 = totally disagree) based on the Unified Theory of Acceptance and Use of Technology (UTAUT model) [41] relating to five dimensions that may impact adoption and use of technology: performance expectancy (four items), effort expectancy (four items), social influence (four items), facilitating conditions (four items) and behavioural intention to use the intervention (1 item). Statements relating to GP nurse attitudes (four items) and self-efficacy (two items) with respect to (their ability) using the intervention were included.

At 6-month follow-up GP nurses were asked about their experiences and satisfaction with the CDMIS and guiding patients; which strategies they used to guide patients (with a high and lower SES), the amount and type of guidance that they had given (e.g. the intensity of the guidance) and how competent they felt in guiding patients with a lower-SES level.

In-depth unstructured interviews were conducted with GP nurses (*n* = 10) to gain deeper insight into their perceptions about the barriers and facilitators of implementing the CDMIs alongside open-ended questions in the questionnaire.

#### Additional factors

Patient socio-demographics included age, gender (male/female), living arrangement (alone, with partner, with partner and children, without partner and with children, with parents, other), marital status (single, married, civil partnership, divorced, widowed) and country of birth. Demographic data collected among GP nurses included age, gender, educational level, work-related factors (e.g. working hours, years of work experience and experience in referring to/ using online interventions).

### Sample size calculation

Aim of the study was to double the participation rate of lower-SES patients from 18 to 36%. Due to a paucity of studies about online psychological interventions offered and guided by primary care professionals, this 18% base rate was based on previous (baseline) participation rates of lower-educated people in studies of online self-help interventions for depression [5, 17] and also takes into account that our definition of participation entails intervention participation rather than trial participation. The difference in expected participation rates had to be tested at α ≤ 0.05 (2-tailed) and a power of (1-β) = 0.80, while accounting for an average cluster size of 6 patients per GP nurse (range 2–14 patients) with an intra-class correlation of 0.02 [42]. This required 114 patients ‘nested’ in 19 GP nurses per condition, or 228 participants and 38 GP nurses in total. The sample size calculation was performed in Stata version 14.2 statistical software package using the clustersampsi-procedure.

### Randomisation

The randomisation of GP nurses was performed by an independent statistician using a computer-generated schedule stratified for neighbourhood SES levels (low, medium, high SES) to balance the distribution of this factor across the two conditions. GP nurses could not be blinded as they knew whether their allocated implementation strategy consisted of specific components aimed at reaching lower-SES patients. Patients were not made aware of the randomisation status of their GP nurse.

### Statistical analysis

#### Sample characteristics and attrition

Descriptive statistics were used to describe the characteristics of the GP nurses and patients at baseline. For attrition analyses see Additional file 2.

#### Main analysis

Analyses were carried out in agreement with the intention-to-treat principle for which logistic mixed models were used to estimate the effects of the implementation strategies on the participation rate of lower SES. This technique accounts for the correlation of data when measurements are ‘nested’ in patients and patients ‘nested’ within GP nurses. Missing values were accounted for using the maximum likelihood method to estimate coefficients. A random intercept model was fitted with an identity covariance structure. The implementation condition was used as a fixed between-groups factor.

### Sensitivity analyses

The main analysis was repeated using a lower cut-off for educational level (MBO-1 instead of MBO-4) to define lower SES. By way of sensitivity analysis, we also explored using more than the median number of CDMI exercise use as an alternative operationalization of participation. We also explored whether health literacy modified the effect of implementation condition on lower SES participation.

#### Analysis of secondary outcomes

Linear mixed models were used to analyse the development of psychological complaints in patients over time (T0, T1 and T2). The interaction between implementation condition and time was added to determine within and between group development of psychological complaints.

Descriptive statistics were used to evaluate the implementation process and interviews were transcribed and analysed with MaxQDA version 18.2.3.

All statistical analyses were performed using SPSS version 25 and Stata version 12.1.

## Results

### Study flow

The study flowchart is depicted in Fig. 1. Two-thirds of GP nurses in the all- SES group and half the GP nurses in the SES-sens group included patients in the trial. The distribution of the two participating regions was balanced across the conditions: Almere (*n* = 7, 58%) and Rotterdam (*n* = 5, 42%) in the SES-sens condition and similarly Almere (*n* = 9, 64%) and Rotterdam (*n* = 5, 36%) in the all-SES condition. The percentage of GP nurses working in a high-SES neighbourhood was slightly higher in the all-SES condition (high *n* = 8 [57%], medium *n* = 3 [21%], low *n* = 3 [21%]) than the SES-sens condition (high *n* = 4 [33%], medium *n* = 4 [33%], low *n* = 4 [33%]). Of the 26 GP nurses who actually included patients, there were 7 pairs of GP nurses who worked in the same GP practice. More patients were included by the GP nurses in the all-SES- condition than in the SES-sens condition (Fig. 1).

### Baseline characteristics

Baseline characteristics of GP nurses and patients are presented in Table 1 and Table 2, respectively. Thirteen GP nurses in the all-SES condition and nine GP nurses in the SES-sens condition completed the baseline questionnaire. All included patients completed the baseline questionnaire. Patients were largely female, born in The Netherlands, employed, and nearly half had a medium educational level. In both groups, the majority (80%) reported quite severe mental health complaints. There were fewer women and fewer cases of stress in the SES-sens condition. For a detailed baseline and attrition analysis see Additional file 2.

**Table 1:** GP nurse characteristics at baseline

Characteristics^a^		**All-SES implementation strategy** ***n* = 13**	**SES-sens implementation strategy** ***n* = 9**
**Sociodemographic and work experience**			
**Gender**	female	12 (92%)	7 (78%)
	male	1 (8%)	2 (22%)
**Age** (in years)		44.4 (9.9)	48.0 (12.8)
**Education level**	Non-academic	8 (62%)	7 (78%)
	Academic	5 (39%)	2 (22%)
**Working hours** (per week)		27.0 (5.8)	29.2 (7.2)
**Number of years’ experience in mental health services**		11.2 (9.6)	18.1 (7.5)
**Number of years’ experience as a GP nurse**		4.3 (2.9)	5.0 (2.3)
**How often have you referred patients to online self-help?**	Often/ very often	7 (54%)	5 (56%)
**Experience with guiding patients using online self-help**	Quite a lot/ a lot	3 (23%)	2 (22%)
**Internet skill level**	Good/ very good	9 (69%)	5 (56%)
**Expectations about guiding patients with a lower SES (using online self-help)**			
**I feel capable of guiding patients with a lower SES level**	Agree/completely agree	10 (77%)	6 (67%)
**I expect that online guided self-help interventions can be effective for patients with a lower SES-level**	Agree/completely agree	6 (46%)	4 (44%)
**Online guided self-help interventions are only suitable for motivated patients**	Agree/completely agree	9 (69%)	9 (100%)
**I feel capable of guiding patients with a lower SES to use online self-help interventions**	Agree/completely agree	9 (69%)	4 (44%)
**UTAUT implementation factors**			
**Performance expectancy**	scale 4–20	6.2 (2.2)	7.1 (2.2)
**Effort expectancy**	scale 4–20	9.8 (1.5)	8.9 (2.1)
**Social influence**	scale 4–20	9.5 (1.8)	9.7 (2.6)
**Facilitating conditions**	scale 4–20	7.6 (2.9)	7.3 (2.5)
**Attitude towards using technology**	scale 4–20	7.2 (2.0)	8.3 (1.7)
**Self-efficacy in using technology**	scale 2–10	3.8 (1.3)	3.9 (1.5)
**Intent to offer the online CDMIs**	scale 1–5	1.2 (0.6)	1.1 (0.3)

^*^ Percentages may not add up to 100% due to rounding. ^a^ Data are mean values (SD) or n (%)

**Table 2:** Characteristics of patients at baseline

		**All-SES implementation strategy** *n* = 135	**SES-sens implementation strategy** *n* = 93
**Sociodemographic factors**			
**Age**		40.6 (14.3)	39.6 (13.7)
**Gender**	Female	103 (76%)	59 (63%)
	Male	32 (24%)	34 (37%)
**Marital status**	Not married	6 (45%)	48 (52%)
	Married/Living with partner	67 (50%)	36 (39%)
	Divorced	7 (5%)	9 (10%)
	Widowed	0 (0%)	0 (0%)
**Country of birth**	The Netherlands	125 (93%)	81 (87%)
	Other	10 (7%)	12 (13%)
**Living arrangement**	Alone	15 (11%)	22 (24%)
	Not alone	120 (89%)	71 (76%)
**Education**	Low	25 (19%)	16 (17%)
	Medium	66 (49%)	44 (48%)
	High	44 (33%)	32 (35%)
**Income**	Above social minimum	123 (91%)	77 (84%)
**Employment**	Employed	105 (78%)	63 (68%)
	Unemployed	3 (2%)	11 (12%)
	Other (e.g. student, pension)	27 (20%)	19 (20%)
**Clinical factors**			
**Duration complaints**	< 1 year	74 (55%)	47 (51%)
	≥ 1 year	61 (45%)	46 (50%)
**Severity complaints**	Low	25 (19%)	19 (20%)
	High	110 (82%)	74 (80%)
**Most troubling symptom**	Sleep	23 (17%)	18 (19%)
	Stress	63 (47%)	27 (29%)
	Worry	49 (36%)	48 (52%)
**Depressive complaints**	(scale 0–24)	12.9 (4.8)	12.3 (5.4)
**Sleep problems**	(scale 0–20)	12.7 (5.1)	11.9 (5.6)
**Stress**	(scale 0–40)	23.5 (5.8)	23.2 (6.4)
**Worry**	(scale 11–55)	40.1 (7.9)	40.3 (8.7)
**Anxiety**	(scale 0–21)	11.4 (4.5)	11.2 (4.8)
**Wellbeing**	(scale 0–100)	25.2 (16.5)	28.6 (19.7)
**Implementation-related factors**			
**Health literacy**	(scale 1–4)	3.0 (0.4)	3.0 (0.4)
**Internet skill level**	Good/very good	122 (90%)	83 (89%)
**I have previous experience with online interventions**	Yes	53 (40%)	32 (34%)
**I expect the online CDMIs will reduce my complaints**	Agree a little/ completely	98 (73%)	62 (67%)
**I expect that I am capable of using the online CDMIs**	Agree a little/ completely	121 (90%)	80 (86%)
**I intend to use the CDMIs in the upcoming four weeks**	Agree a little/ completely	122 (90%)	81 (87%)

^*^ Percentages may not add up to 100% due to rounding. ^a^ Data are mean values (SD) or n (%)

### Primary outcome

The intra-class correlation for the primary outcome approached zero for the model that included the implementation condition as a fixed factor in the mixed model (ICC < 0.001). The percentage of patients with lower SES was high in both groups (Table 3). However, there were significantly more patients with lower-SES in the all-SES group than the SES-sens group (Table 3).

**Table 3:** Analysis of the primary outcome

	All-SES strategy *N* = 130	SES-Sens strategy *n* = 85	OR^a^ (95% CI) intervention vs control	z	p	OR ^b^ (95% CI) intervention vs control	z	p
**Lower SES participation,** yes (%)	75 (58%)	37 (44%)	0.56 (0.35 to 0.98)	-2.02	.043	0.43 (0.22 to 0.81)	-2.58	.010
**Alternative definitions of lower SES participation,** yes (%)								
Education level defined as MBO 1 or lower	20 (15%)	11 (13%)	0.83 (0.30 to 2.26)	-0.37	.709	0.65 (0.20 to 2.08)	-0.73	.464
Exercise use defined as 12 or more exercises	48 (36%)	18 (20%)	0.44 (0.24 to 0.83)	-2.56	.010	0.35 (0.17 to 0.73)	-2.80	.005

^a^ Crude model

^b^ Model adjusted for: gender (patient), GP nurse self-efficacy in using technology and GP nurse previous referrals to online self-help

We examined the various components of the primary outcome. The main difference in the components of the primary outcome is the percentage of patients who had at least 1 face-to-face session with the GP nurse which is higher in the all-SES group than in the SES-sens group (Table 4).

**Table 4:** Distribution of the components of the primary outcome across conditions

	All-SES strategy *n* = 135	SES-Sens strategy *n* = 93
**Criterion 1: Lower SES**		
MBO-4 or lower	91 (67%) 0 missing	60 (65%) 1 missing
Unemployed and living in a low-ses neighbourhood	0 (0%) 0 missing	6 (7%) 0 missing
Family income below social norm and living in a low-ses neighbourhood	3 (2%) 0 missing	9 (10%) 1 missing
**Criterion 2: at least 1 face-to-face session with the GP nurse**	114 (92%) 11 missing	56 (72%) 15 missing
**Criterion 3: engaging in at least 2 exercises**	124 (92%) 0 missing	83 (89%) 0 missing
**Meeting all criteria for primary outcome**	75 (58%)	37 (44%)

In the all-SES condition two GP nurses were responsible for enrolling 55 of the 130 patients (42%) and for 35 of the 75 lower-SES participants (47%). To find out to what extent these two GP nurses influenced the outcomes, analyses were also run without them, resulting in (approximately) the same magnitude of the effect (adjusted OR 0.48, 95% CI, 0.22 to 1.03, *p* = 0.059). The lack of significance was likely due to reduced power after excluding 55 cases.

#### Alternative definitions of the primary outcome

Re-defining the educational level component of our lower SES outcome more stringently as having an MBO-1 education level or lower resulted in lower percentages of participation rates in both conditions, with no significant differences between the conditions (Table 3). When we defined ‘using the intervention’ as the median number of logged exercises in the total sample (median = 12) or better, this resulted in lower participation rates in both conditions. However, there was a significant difference between the groups with a higher participation rate in the all-SES group (Table 3).

#### Effect modification of health literacy

We examined whether baseline patient health literacy skills modified intervention effects. The interaction term between condition and health literacy was not significant (OR 2.56, 95% CI 0.66 to 9.83, *p* = 0.172).

### Secondary outcomes

#### Psychological complaints and wellbeing

Patients had quite severe complaints at baseline (see Table 1). There was a significant decrease in depressive complaints (PHQ-8) among patients in both groups at T1 and T2 as compared to T0 (Table 5) but there was no significant difference between the two groups. The same pattern was found for the outcomes on worry (PSWQ), anxiety (GAD-7) and wellbeing (WHO-5). However, the difference between groups for sleep over time was *p* = 0.05 (JSEQ, likelihood ratio test chi square, 2df, = 5.88, *p* = 0.05).

**Table 5:** Observed means of psychological complaints and within group estimated changes at T1 and T2 compared to baseline (T0)

	All-SES implementation strategy				SES-sensitive implementation strategy			
	Mean (SD)	Estimate^a^ (95% CI)	z-value	p	Mean (SD)	Estimate^a^ (95% CI)	z-value	p
**Depression (PHQ-8)**								
T0	12.9 (4.8)	reference			12.3 (5.4)	reference		
T1	7.1 (4.0)	-5.88 (-7.11 to -4.67)	-9.47	.000	7.8 (5.0)	-4.41 (-5.93 to -2.89)	-5.67	.000
T2	6.5 (3.6)	-6.22 (-8.01 to -4.43)	-6.83	.000	5.6 (3.1)	-6.67 (-8.59 to -4.74)	-6.77	.000
**Sleep (JSEQ)**								
T0	12.7 (5.1)	reference			11.9 (5.6)	reference		
T1	6.9 (4.5)	-5.92 (-7.33 to -4.50)	-8.21	.000	9.1 (6.3)	-3.07 (-4.84 to -1.31)	-3.41	.001
T2	6.3 (3.4)	-6.52 (-8.59 to -4.45)	-6.17	.000	6.1 (4.3)	-6.10 (-8.33 to -3.86)	-5.35	.000
**Stress (PSS)**								
T0	23.5 (5.8)	reference			23.2 (6.4)	reference		
T1	16.8 (6.4)	-6.94 (-8.67 to -5.19)	-7.81	.000	16.2 (7.1)	-6.41 (-8.59 to -4.24)	-6.14	.000
T2	16.1 (6.1)	-8.04 (-10.59 to -5.48)	-6.17	.000	14.1 (7.1)	-8.64 (-11.39 to -5.88)	-6.14	.000
**Worry (PSWQ)**								
T0	40.1 (7.9)	reference			40.3 (8.7)	reference		
T1	34.8 (7.9)	-6.03 (-7.87 to -4.19)	-6.42	.000	33.6 (9.3)	-5.27 (-7.57 to -2.96)	-4.48	.000
T2	33.2 (5.9)	-7.37 (-10.06 to -4.67)	-5.36	.000	27.6 (7.4)	-11.14 (-14.05 to -8.23)	-7.50	.000
**Anxiety (GAD-7)**								
T0	11.4 (4.5)	reference			11.2 (4.8)	reference		
T1	6.0 (3.9)	-5.21 (-6.35 to -4.06)	-8.91	.000	5.8 (4.8)	-4.80 (-6.23 to -3.37)	-6.57	.000
T2	5.9 (4.0)	-5.31 (-6.98 to -3.63)	-6.19	.000	4.4 (4.9)	-6.37 (-8.18 to -4.55)	-6.88	.000
**Wellbeing (WHO-5)**								
T0	25.2 (16.5)	reference			28.6 (19.7)	reference		
T1	45.2 (20.0)	19.78 (14.39 to 25.18)	7.19	.000	50.0 (23.0)	19.04 (12.29 to 25.79)	5.53	.000
T2	48.7 (21.5)	23.49 (15.57 to 31.40)	5.81	.000	60.3 (22.8)	27.76 (19.21 to 36.30)	6.37	.000

^a^ All estimates are adjusted for age, health literacy, expected effectiveness of CDMIs and intention to use the CDMIs at baseline

#### Implementation process indicators

##### Patients -Use of CDMIs

According to automated CDMI activity log, patients completed a mean of 13 unique CDMI exercises in the all-SES condition and 12 in the SES-sens condition.

Questions on utility and satisfaction (T1) were completed by 28 patients in the SES-sens condition and 44 in the all-SES condition. In the SES-sens condition, 24 patients (86%) reported that they used the online CDMIs of whom 12 (50%) reported spending an average of 30 min or more a week and none spent more than 2 h a week on the CDMIs. In the all-SES- group 39 (89%) reported use of CDMIs and the majority (54%, *n* = 21) spent at least 30 min or more and 5% (*n* = 2) 2 h or more a week on the CDMIs.

##### Patient Satisfaction with the CDMIs

**Fig. 2:**
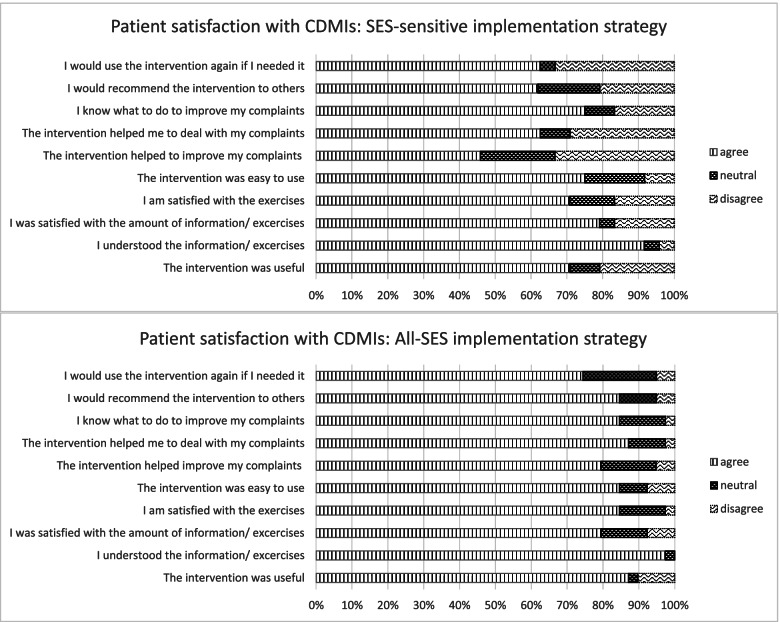
Patient satisfaction with the CDMIs for the all-SES and SES-sensitive implementation conditions

Overall satisfaction with the CDMIs was moderate to high. The overall satisfaction score (on a scale 1 to 10) was higher in the all-SES condition (*n* = 44, Median = 8, Mean = 6.5, SD = 1.5) than in the SES-sens condition (*n* = 24, Median = 7 Mean = 6.5, SD 1.5). Figure 2 shows the ratings on each of the satisfaction items by condition. A pattern of moderate to high satisfaction can be discerned across the various topics, with higher satisfaction ratings in the all-SES condition.

##### Patient Satisfaction with guidance from GP nurses

**Fig. 3:**
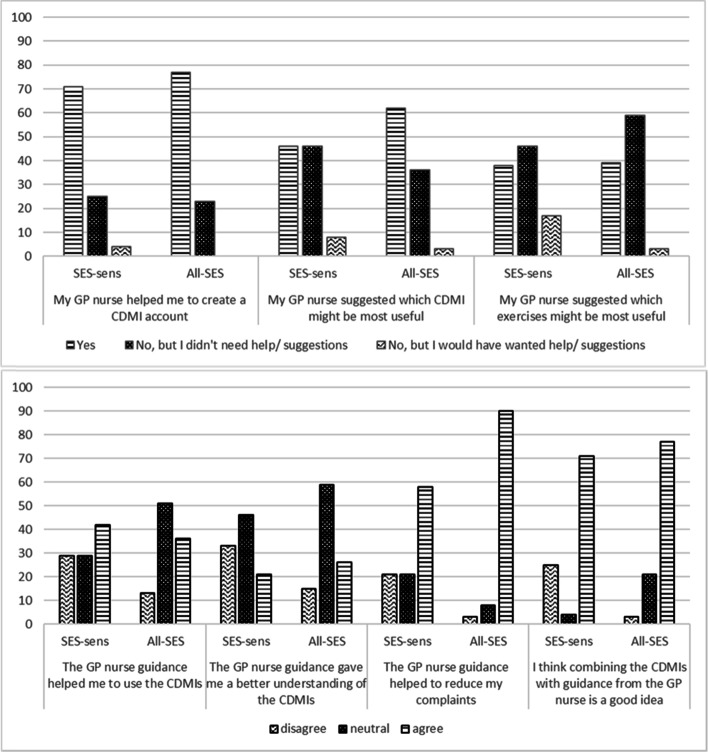
Patients’ satisfaction (%) with GP nurse guidance in the SES-sensitive (*n* = 24) and all-SES (*n* = 45) implementation groups

Overall satisfaction with guidance provided by GP nurses was moderate to high. The all-SES group gave a higher satisfaction rating (*n* = 45; Median 8, Mean = 8.2, SD 1.0) than the SES-sens group (*n* = 24, Median = 7, Mean = 6.7, SD 2.3). The majority of patients indicated that they were satisfied with the amount of contact they had with their GP nurse, albeit more so in the SES-sens group (79%) than the all-SES group (69%). Figure 3 shows the ratings of patients on each of the satisfaction items. These results show a similar pattern of moderate to high satisfaction across the various topics, with higher satisfaction ratings in the all-SES group.

##### Patients-Barriers and facilitators of implementation

Most patients stated that once they were logged in, the website was easy to use and clearly structured, although others mentioned that it would help if a GP nurse would show all the options available in the CDMIs. Also, participants appreciated the CDMIs for the variety of exercises and that they could recognize themselves in the problems mentioned in the exercises.

Barriers encountered by patients for using the CDMI’s were often related to technological issues. For example, logging on to the website was sometimes experienced as difficult and patients would have preferred a smartphone application. Further, some exercises were experienced as too repetitive or not challenging enough, and some were not practical or concrete enough. Suggestions were to add more depth to existing exercises, and add more exercises. Patients suggested that GP nurses could provide more guidance or check in with patients through email or phone. Patients in the SES-sens group mentioned that more guidance from the GP-nurses would have helped to stay motivated to continue exercising.

##### Satisfaction with intervention and materials among GP nurses, and perceived suitability for lower SES patients

**Fig. 4:**
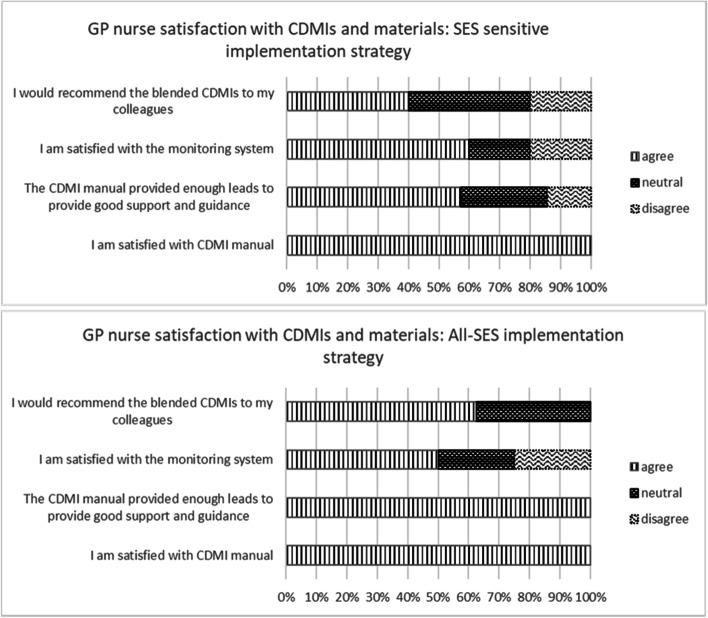
GP nurse satisfaction with the CDMIs and implementation materials for the all-SES and SES-sensitive implementation conditions

Six GP nurses (50%) completed questions on process indicators in the SES-sens condition and 8 (57%) in the all-SES condition. According to the overall satisfaction score GP nurses in the SES-sens condition were moderately satisfied with the CDMIs (Median = 7, Mean = 6.7, SD = 1.0) and a little more satisfied in the all-SES condition (Median = 7.5, Mean = 7.5, SD = 0.9). Figure 4 shows the ratings of GP nurses on each of the satisfaction items. They were generally positive about the guidance manual. However, only half the GP nurses in both conditions were satisfied with the monitoring system and GP nurses gave mixed ratings with respect to recommending the blended CDMIs to their colleagues.

Two GP nurses (33%) in the SES-sens condition and four (50%) in the all-SES condition indicated that online interventions would be effective for patients with a low education level. Three GP nurses in the SES-sens condition and one in the all-SES condition felt that the educational level of their patients was too low to participate in the CDMIs. At least half of the GP nurses in both groups agreed that the online CDMIs were relevant and suitable for their patients and their patients often wanted to start the intervention once it was offered.

##### Adoption of implementation (sub)strategies among GP nurses

**Fig. 5:**
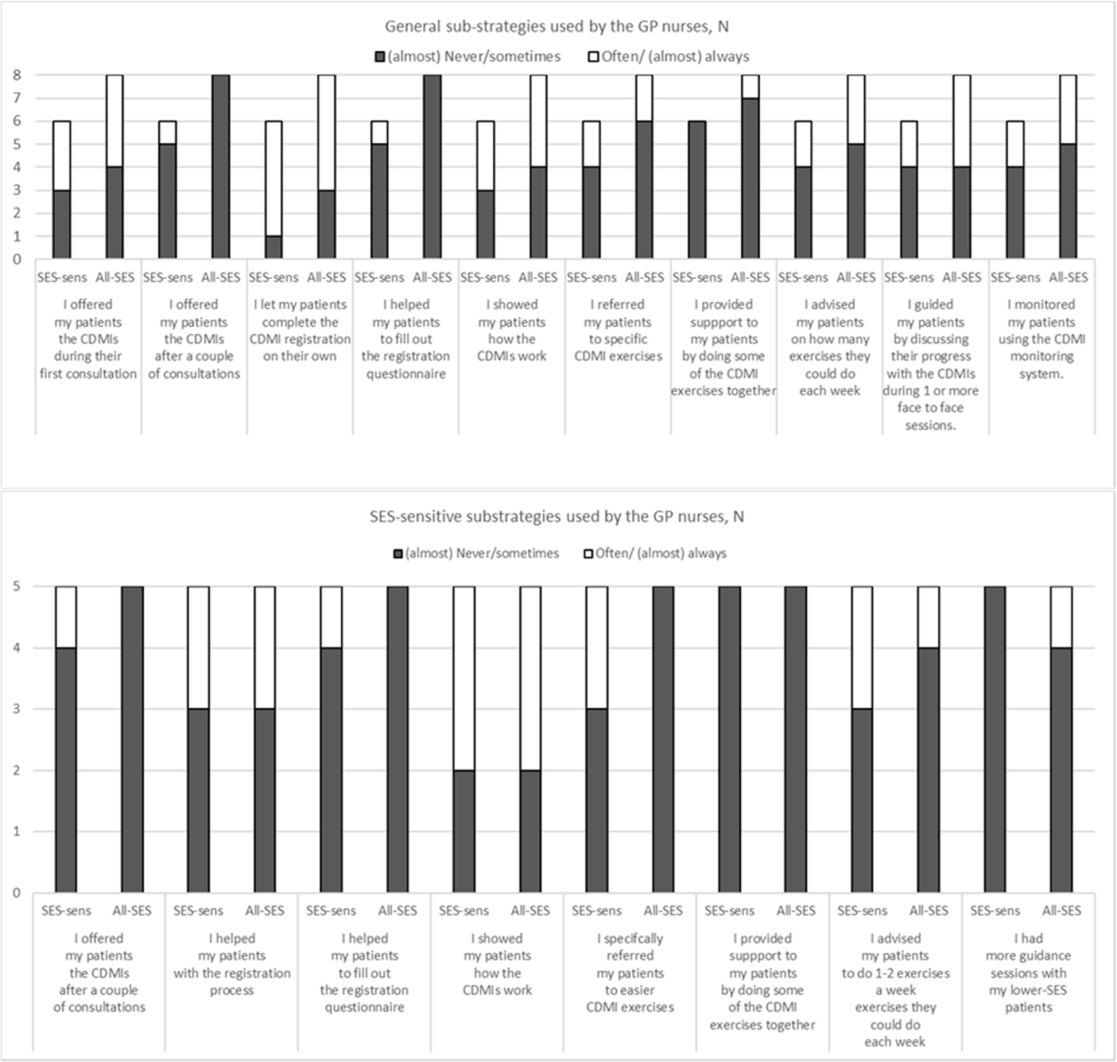
Implementation sub-strategies used by the GP nurses in the all-SES and SES-sensitive implementation conditions

Many of the implementation strategies were not applied by GP nurses in either condition (Fig. 5). The SES-sensitive sub-strategies were not used regularly in the SES-sens group. In fact, some SES-sensitive strategies were used in equal measure in the all-SES group, although the GP nurses did not receive specific training or instruction to implement these strategies.

##### Perceived implementation barriers and facilitators among GP nurses

GP nurses in both conditions mentioned that easy access and user friendliness of the website helped guide participants through the exercises. They praised the website for its flexibility and simplicity. They mentioned that the exercises related well to the patients’ complaints.

Barriers encountered by GP nurses in both conditions included that not all GP nurses received additional time from their employer to learn how to work with the CDMIs. Also, from the interviews, we discerned a tendency from the GP nurses to view the CDMIs less as a central feature of their treatment, but only as a supplement to it. This also translated into the nature of face-to-face guidance provided to the patients in their care. None of the scheduled GP nurses’ sessions were solely focused on guiding patients with the CDMIs. Guidance usually only consisted of asking patients if they did any exercises and if they had questions. GP nurses noted that the monitoring system did not provide enough insight into what the patients had done in the CDMIs or that the information in the system was unclear. This impeded the use of the CDMIs as an integral part of treatment.

Some GP nurses thought the exercises were too complicated for people with lower SES and too easy for people with higher SES. Furthermore, GP nurses mentioned that the registration process for the participants was too extensive and therefore participants dropped out before starting the CDMIs. They noted that efforts to simplify the intervention for people with less internet skills would provide a more positive experience as well as making it accessible for use on a smartphone.

In addition, GP nurses in the SES-sens group had difficulty recognising the lower SES group. They mentioned that the definition provided of lower SES was not clear enough. GP nurses in the SES-sens group noted that people (with higher or lower SES) do not always own a pc, and that patients with lower SES had more urgent problems to address (housing, financial issues, etc.) than doing the CDMIs. Additionally, they noted that people with a lower SES might speak a different language and need more visual explanations of the exercises instead of written explanations. Lastly, they noticed that people with lower SES were less likely to stay motivated to follow through with the CDMIs, and more prone to discontinuing the usage of the CDMIs and the guidance by the GP nurse as soon as they felt some improvement in their mental health.

## Discussion

In this study, we aimed to evaluate whether a SES-sensitive implementation strategy as compared to a strategy without SES-sensitive elements, improves the participation rate (i.e. reach) of patients with a lower SES in the blended online CDMIs in primary care. Overall, there was a higher proportion of patients with a lower SES in both groups than expected at the start of the study. We expected 18% of lower SES participants and aimed to double this to 36% by using a SES-sensitive implementation strategy, but our study showed a 44% of lower SES participants in the SES-sens condition and a 56% participation rate in the all-SES condition. This difference was statistically significant in favour of the all-SES group and remained significant in a series of sensitivity analyses (e.g. when adjusted for covariates). From a public health perspective, it is positive that such a high percentage of lower-SES patients was reached. Targeting patients with a lower SES in primary care by GP nurses seems to be a useful approach, as our previous trial had a lower intervention reach (28%) among patients with a lower SES when the intervention was delivered in an unguided self-help format [17]. We also found that patients in this study used more unique exercises on average than in the previous RCT, approximately 12 versus 8, respectively [43]. This aligns with the idea that guidance may be beneficial to engagement with online interventions [12].

The main driver of the between-group difference in outcome (participation rate of lower-SES patients) was due to the fact that less guidance was provided by the GP nurses in the SES-sens condition: if guidance was disregarded from the main outcome there would not be any notable difference between the conditions. Moreover, we controlled for possible confounders between conditions, but this did not explain the difference in guidance. The all-SES condition was generally more positive about the implementation process than the SES-sens condition, and GP nurses found the definition of lower-SES in our study unclear and therefore had difficulty recognising lower-SES patients. Taken together, we hypothesise that the SES-sensitive strategy could have been perceived as confusing or overly complicated by the GP nurses which impacted adversely on their ability to guide lower-SES patients. Although the GP nurses provided less guidance than instructed, especially in the SES-sens condition, this did not translate into differences in the use of the CDMIs or their impact on the reduction of complaints between the conditions. As guidance has been related to increased adherence to online interventions [12], our findings raise the question about the type and frequency of guidance that is needed to impact adherence and effectiveness. The less substantial and frequent guidance is, the more scalable e-health interventions can be [44, 45]. Our study suggests that the act of explaining and offering the CDMIs in primary care may be an effective strategy to engage lower SES patients with an online intervention for depression prevention.

We also found that the CDMIs could be used by patients with an MBO-1 educational level or lower (rather than an MBO-4 level or lower), though the reach of this group was relatively small in this this study. Various issues were mentioned as barriers: practical (e.g. lack of easy internet access or access via smartphone), content-related (e.g. difficulty of exercises, needing help with social/ financial issues) and preference-related (e.g. wanting to discuss problems with GP-nurse, lack of motivation to use e-health) in our study. At the start of the study, we surmised that guidance would help to overcome some of these barriers, yet this solution proved insufficient.

### Considerations

A limitation of this study is that both study conditions entailed active implementation strategies, thus there was no non-active comparison group. Also, although the CDMIs were developed as an intervention for depression prevention, participating patients had quite high levels of complaints at baseline, higher than in the RCT investigating the effectiveness of the unguided CDMIs [17]. This study was developed to evaluate implementation strategies, not to evaluate improvement of mental health; we therefore cannot make causal inferences with respect to the symptom decreases in both groups.

Although various potential differences between the implementation groups were evaluated and controlled for, it cannot be ruled out that unmeasured differences may have influenced the findings of our study.

Another aspect concerns the actual implementation level. In their review, Vis et al. [46] noted that when implementing e-mental health in routine care, important factors to consider are acceptance, expectations and preferences of patients and professionals, appropriateness of the intervention in addressing the mental health problem, and the availability, reliability and interoperability of the technology. These are all factors that were considered at the start of this study and brought to the attention of the participating GP nurses during their implementation training, and yet the actual implementation of the CDMIs was far from optimal as evidenced by the limited use of the sub-strategies (including guidance) in both groups. Our findings show that future implementation efforts should include more integration of the e-health technology within clinical practice, such as providing better insight into patient progress using the monitoring system. It is also of importance to find (even) more optimal solutions to encountered barriers in daily practice of GP nurses and patients (e.g. time-constraints, technological difficulties, easy access using smartphone technology and training).

### Future directions

The implementation of e-health interventions for mental health problems is receiving increasing attention in the literature due to the importance of increasing their uptake and adherence [47]. However, experimental implementation research is scarce [47]. Our study contributes to this scarce evidence base. A prior study successfully increased the acceptance of internet-based depression prevention interventions among primary care patients as a means to increase uptake [48]. In our study, we implicitly aimed to increase acceptance through training GP nurses and by offering (lower-SES) patients low-threshold access to e-health in primary care with guidance from their GP nurse. In future research, it may be valuable to add explicit acceptance-enhancing interventions to the implementation strategies we developed. Moreover, we echo the call for more experimental research into effective implementation strategies [47], especially when it comes to reaching high risk target groups as they stand to benefit most from appropriate preventive intervention for mental health problems.

## Conclusions

The implementation of the online CDMIs in primary care with guidance from a GP nurse can improve participation rates among patients with a lower socioeconomic status compared to our a priori expected participation rate based on previous findings [19]. Contrary to expectations, the participation rate was higher using an implementation strategy not explicitly directed at guiding lower SES than a tailored SES-sensitive strategy. Overall implementation of the CDMIs was sub-optimal as there was limited use of the implementation sub-strategies (including guidance) in both groups among GP nurses. From a public health point of view, it is positive that a substantial number of patients with a medium to low education level were reached with the CDMIs using minimal guidance. Implementing e-health in regular primary care may be an important step for increasing the reach of depression prevention and warrants further research into how implementation strategies can be optimised in primary care settings which are an important entry point for all users in a given health system.

## Supplementary Information

**Additional file 1.** CONSORT Extension for Cluster Trials 2012 Checklist.

**Additional file 2.** Baseline and attrition analysis.

## Data Availability

The study data are available from the corresponding author on reasonable request.

## Abbreviations

CDMI: Complaint-Directed Mini-Interventions
FCCHL: Functional Communicative and Critical Health Literacy scale
GAD-7: 7-Item Generalized Anxiety Disorder Scale
GP: General practitioner
GP Nurse: General practice nurse
HADS: Hospital Anxiety and Depression Scale
JSEQ: Jenkins Sleep Evaluation Questionnaire
MBO: Intermediate vocational education (Dutch: Middelbaar Beroepsonderwijs)
PHQ-8: 8-Item Patient Health Questionnaire
PHQ-9: 9-Item Patient Health Questionnaire
PSS-10: 10-Item Perceived Stress Scale
PSWQ: Penn State Worry Questionnaire
SES: Socioeconomic status
SES-sens: SES-sensitive
UTAUT: Unified Theory of Acceptance and Use of Technology
WHO-5: 5-Item World Health Organization Well-Being Index
WSQ: Web Screening Questionnaire
