# Supplementary material for: Comparing a ses-sensitive and an all-ses implementation strategy to improve participation rates of patients with a lower socioeconomic background in a web-based intervention for depressive complaints: a cluster randomised trial in primary care
Source: BMC Prim Care. 2022 Aug 11;23:205. doi: 10.1186/s12875-022-01793-w (PMC9367024; doi:10.1186/s12875-022-01793-w)
Supplement: Supplementary file 2 — Additional file 2. Baseline and attrition analysis. [file 12875_2022_1793_MOESM2_ESM.pdf]

ADDITIONAL FILE 2 BASELINE AND ATTRITION ANALYSIS

Baseline and attrition analysis: method

Prognostically important factors relating to baseline imbalances and possibly selective attrition were identified using multivariate logistic models using a backward-stepping procedure. Only factors that were related to baseline imbalances or selective attrition in univariate models (using a liberal p-value of ≤ 0.20) were included in multivariate models. Variables that were related to attrition in these models, using a p-value of ≤ 0.2, were checked to identify whether they were confounders of the primary outcome (i.e. they changed the estimate by at least 10%).

Baseline and attrition analysis: results

Loss to follow-up on the primary outcome was 8% overall and was higher in the SES-sens group (13%) than the all-SES group (4%) (Figure 1). Factors that were multivariately related ($p \leq 0.2$) to loss to follow-up at the GP nurse level were more self-efficacy, having previously referred to the CDMIs, and the GP practice being in a predominantly low SES neighbourhood. Multivariate predictors of dropout at the patient level were country of birth other than The Netherlands, a lower health literacy level, and less intention to use the CDMIs.

Attrition of patients on the secondary psychological outcomes was 68% at T1 and 85% at T2. Factors that were multivariately related ($p \leq 0.2$) to psychological complaints were a younger age, a lower expectancy of CDMI effectiveness, a lower level of health literacy level, and less intention to use the CDMIs.

All of the above factors were explored in the analysis of potential confounders. The following factors were also considered as potentially important due to (slight) baseline imbalances: GP nurses' attitude towards using technology and years of experience in mental health care, and patients' level of

wellbeing, gender, country of birth and type of complaint. See **Tables 3 and 5** for the final adjusted analyses.
